# Time to Endoscopy in Patients with Colorectal Cancer: Analysis of Wait-Times

**DOI:** 10.1155/2016/8714587

**Published:** 2016-04-06

**Authors:** Renée M. Janssen, Oliver Takach, Estello Nap-Hill, Robert A. Enns

**Affiliations:** ^1^Department of Medicine, University of British Columbia Faculty of Medicine, Vancouver, BC, Canada V5Z 1M9; ^2^Division of Gastroenterology, St. Paul's Hospital, University of British Columbia Faculty of Medicine, Vancouver, BC, Canada V6Z 2K5; ^3^Department of Medicine, Division of Gastroenterology, St. Paul's Hospital, Vancouver, BC, Canada V6Z 2K5

## Abstract

*Objective*. The Canadian Association of Gastroenterology Wait Time Consensus Group recommends that patients with symptoms associated with colorectal cancer (CRC) should have an endoscopic examination within 2 months. However, in a recent survey of Canadian gastroenterologists, wait-times for endoscopy were considerably longer than the current guidelines recommend. The purpose of this study was to evaluate wait-times for colonoscopy in patients who were subsequently found to have CRC through the Division of Gastroenterology at St. Paul's Hospital (SPH).* Methods*. This study was a retrospective chart review of outpatients seen for consultation and endoscopy ultimately diagnosed with CRC. Subjects were identified through the SPH pathology database for the inclusion period 2010 through 2013. Data collected included wait-times, subject characteristics, cancer characteristics, and outcomes.* Results*. 246 subjects met inclusion criteria for this study. The mean wait-time from primary care referral to first office visit was 63 days; the mean wait-time to first endoscopy was 94 days. Patients with symptoms waited a mean of 86 days to first endoscopy, considerably longer than the national recommended guideline of 60 days. There was no apparent effect of length of wait-time on node positivity or presence of distant metastases at the time of diagnosis.* Conclusion*. Wait-times for outpatient consultation and endoscopic evaluation at the St. Paul's Hospital Division of Gastroenterology exceed current guidelines.

## 1. Introduction

Colorectal cancer (CRC) is the third most common malignancy and the second most common cause of death from cancer in Canada [1]. Approximately 21,100 patients are diagnosed each year with colon cancer in Canada [1]. While twenty-five percent of colon cancer cases occur in individuals with risk factors such as a family history of gastrointestinal malignancy or longstanding inflammatory bowel disease, the majority of cases occur in individuals with no clear predictable risk factors besides age [2].

The Canadian Association of Gastroenterology Wait Time Consensus Group recently published recommended guidelines for medically appropriate maximal wait-times for consultation and procedures by a digestive disease specialist [3]. Patients who present with iron-deficiency anemia, a change in bowel habits, bright red blood per rectum (BRBPR), and positive FOBT or FIT should have an endoscopic procedure within 2 months [3]. Patients who have a high likelihood of malignancy, either on imaging or physical exam, should be evaluated and endoscopically assessed within 2 weeks [3].

A number of studies have evaluated wait-times for patients referred for diagnosis and eventual treatment of colorectal cancer. A Manitoba study of 2552 patients found that the overall health system wait-time from initial referral to first treatment of colorectal cancer increased from 61 days in 2001 to 95 days in 2005; the majority of this increase could be attributed to longer wait-times for diagnostic procedures [4]. Leddin et al. conducted a survey of Canadian gastroenterologists, who were asked to report on wait-times for new consultations and endoscopy procedures over a sampling period of one week in 2012; the average wait-time from referral to endoscopy for fecal occult blood positivity was 105 days and 97 days for iron-deficiency anemia [5]. The overall median wait-time was 92 days from referral to consultation, 55 days from consultation to procedure, and 155 days in total from referral to procedure [5]. There was no change in total wait-time from referral to procedure between the 2012 and prior 2008 study [5]. Wait-times for consultation and diagnostic procedures in Canada therefore have continued to exceed current guidelines despite adequate documentation of this substandard process.

The goal of this study was to evaluate the local wait-times at the Pacific Gastroenterology Associates (PGA) office, which is comprised of the ten gastroenterologists (GIs) members of the Division of Gastroenterology at St. Paul's Hospital (SPH) in Vancouver, British Columbia. Specifically, we were interested in the time to initial consultation and endoscopy in patients who were ultimately diagnosed with CRC in the period from 2010 through 2013 and whether wait-times were in accordance with current guidelines.

## 2. Methods

### 2.1. Databases

Patients were identified via the St. Paul's Hospital pathology database, which houses all the reports for all specimens submitted to the Pathology Department at SPH. A natural language search of the word “adenocarcinoma” in the final diagnosis section of the pathology report limited to specimens labeled as “colon” was conducted during the time period January 1, 2010, through December 31, 2013. This period was chosen as it was prior to the implementation of the new colorectal cancer screening program, which commenced in November 2013.

The resultant list was cross-referenced against the Electronic Medical Record (EMR) of the offices of the Pacific Gastroenterology Associates to identify those patients who had been seen by the gastroenterologists of SPH.

For those subjects meeting the inclusion criteria as described below, data was obtained via the EMR of the PGA office as well as the pathology report.

### 2.2. Inclusion/Exclusion Criteria

Subjects were included if they had been referred to and subsequently seen by the SPH gastroenterologists and had a pathologic diagnosis of colorectal cancer via either surgical or endoscopic specimens reviewed at St. Paul's Hospital. Specifically, patients were included if they were referred to the SPH gastroenterologists and seen as outpatients for consultation and endoscopy, with the eventual outcome being a diagnosis of CRC. Patients with known inflammatory bowel disease were excluded given that they are screened more closely than the general population for colorectal cancer. Our population of interest was diagnostically undifferentiated patients who were seen nonurgently in the outpatient setting. Patients were therefore excluded if they were seen either as inpatients or in the Emergency Department at SPH, as they were generally seen as urgent cases. Patients who were enrolled in the Colon Check Pilot program (the pilot predecessor to the current Colon Cancer Screening Program) were also excluded as they were managed differently compared to the general wait list. Patients that had a mass on rectal/physical examination or a high suspicion of malignancy based on diagnostic imaging at the time of referral were also excluded as their procedures were expedited.

### 2.3. Ethics

The authors obtained approval from the UBC-Providence Health Care Research Institute to conduct the retrospective chart review as described herein on December 30, 2013.

### 2.4. Data Extraction

For patients meeting inclusion criteria, the following data were extracted: (1) patient sex and age at time of the pathology result; (2) initial date of receipt of referral to one of the SPH gastroenterologists; (3) referral source; (4) date when patient was first seen by a gastroenterologist; (5) symptoms at presentation; (6) date of first colonoscopy; (7) date of definitive colonoscopy, if multiple colonoscopies were required; (8) reason for multiple colonoscopies, if required; (9) date of tissue diagnosis; (10) date of referral to a surgeon; (11) date when patient was first seen by a surgeon; (12) date of surgery, if required; (13) whether the subject had preoperative chemotherapy or radiation therapy; (14) nodal status at the time of pathologic diagnosis; and (15) presence of distant metastases at the time of diagnosis as determined by staging computed tomography (CT) scan.

Two investigators independently reviewed a subset of subject data to ensure interobserver agreement.

### 2.5. Data Analysis

We defined the wait-times from the initial referral, as follows:

1. Referral to office visit: time to first GI consultation from date of receipt of the referral at the PGA office.
2. Referral to first endoscopy: time to first endoscopy from date of receipt of referral.
3. Referral to definitive endoscopy: time to definitive endoscopy from date of receipt of referral.
4. Definitive endoscopy to surgeon: time from definitive endoscopy to first office visit with a surgeon.
5. Surgeon to surgery: time from office visit with a surgeon to date of surgery.
6. Total wait-time: time from receipt of initial referral to surgery (patients who had preoperative chemo- or radiotherapy are excluded).

Fisher exact or *χ* ^2^ tests were used to assess associations between wait-time, node positivity, and presence of distant metastases. Analysis of wait-times by signs and symptoms was via ANOVA. All tests were 2-sided. A value of* p* < 0.05 was considered to be statistically significant.

## 3. Results

### 3.1. Excluded Patients

Cross-referencing the list of patients generated by the natural language search of the pathology database as described above against the PGA office EMR resulted in a list of 592 subjects. Of these, subjects were excluded for the following reasons: 60 subjects were never actually seen by the SPH gastroenterologists (empty EMR charts or referred but never seen for a variety of reasons); 129 subjects were seen urgently as inpatients at SPH or, rarely, in a private hospital by private pay; 17 subjects were referred or seen outside of the inclusion period as described above; 44 subjects were seen urgently because of a high suspicion of malignancy on either imaging or physical exam at the time of referral; 47 subjects with CRC were referred to the SPH gastroenterologists for reasons other than screening or diagnostic lower endoscopic study, for example, for endoscopic ultrasound to aid in staging of a known rectal cancer; 12 subjects had known inflammatory bowel disease; 19 subjects were seen under the auspices of the Colon Check Pilot program; 6 subjects did not have colorectal cancer (e.g., high grade dysplasia only or adenocarcinoma arising from extra-colonic tissue such as the pancreas); and 13 subjects were lost to follow-up, did not show for their initial consultation or colonoscopy appointment, or initially declined colonoscopy resulting in a delay of diagnosis.

### 3.2. Patient and Cancer Characteristics

There were a total of 246 subjects who met inclusion criteria for this study. Of these, the mean age was 66, and 41% were female. Family physicians were by far the most common referral source, accounting for 95% of referrals. The majority of patients had alarm symptoms at the time of referral. See Table 1 for a summary of patient and cancer characteristics.

The results of surgical pathology demonstrated that 60% of subjects had distal disease, with CRC in either the sigmoid colon or rectum. Five subjects had two primaries at the time of diagnosis of CRC. Thirty-eight percent of subjects had metastatic disease identified in resected lymph nodes. Distant metastatic disease as identified on the staging CT (chest, abdomen, and pelvis) was present in 8% of patients and absent in 87%; in the remaining 5%, either the information was not available or the results were equivocal (e.g., lymphadenopathy or liver lesions present but indeterminate as to whether they represented metastatic disease). See Table 1 for further details regarding cancer characteristics.

One colonoscopy was required for definitive diagnosis in most cases (216 or 88%); 2 lower endoscopy procedures were required in 27 subjects, and more than 2 lower endoscopy procedures were required in only 2 subjects. Reasons for the requirement of a second endoscopy procedure were most commonly due to poor prep, tattooing of the polypectomy site for surgical preparation, or completion of endoscopy when the first study was limited, for example, as with flexible sigmoidoscopy.

### 3.3. Wait-Times

The mean time from receipt of referral to office consultation with a gastroenterologist was 63 days. The mean time from receipt of referral to first lower endoscopy study was 94 days; 102 of 246 subjects (41%) had their first endoscopic procedure within 60 days (guideline benchmark). The mean time from receipt of referral to definitive lower endoscopy study was 97 days. We also analyzed the subset of patients (*N* = 225) who had alarm signs or symptoms at the time of referral (BRBPR, anemia, positive FIT/FOBT, or change in bowel habits); these patients waited a mean of 86 days from time of referral to first endoscopy. There was no effect of wait-time by sign or symptom type (*p* = 0.901). However, there was an effect of presence of signs/symptoms on wait-time, such that those 21 subjects who had no signs or symptoms of CRC at the time of referral waited longer for endoscopy (median 176 days, range 134–213 days) than those subjects who had one or more signs/symptoms (median 70 days, range 35–115 days) (*p* < 0.001).

**Figure 1:**
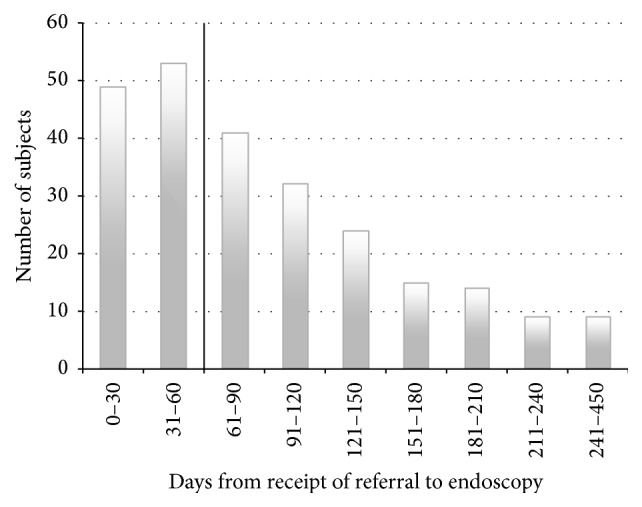
A frequency histogram of wait-time in days from receipt of referral to endoscopy in all included subjects (*N* = 246) is shown in the figure. Wait-times are shown in 30-day intervals with the exception of the last bar (241–450 days). The 60-day benchmark appears as a vertical line at the 60-day mark. 102 of 246 subjects (41%) had their first endoscopic procedure within 60 days; wait-times ranged from 1 to 428 days.

Total wait-time from referral to surgery was 146 days. See Table 2 for a summary of the wait-times from receipt of referral through to surgery. Patients who had a significant delay to surgery, for example, such as those who received presurgery chemotherapy or radiation therapy, were excluded from any analysis that included the time to surgery. A histogram representation of time to first endoscopy in all subjects is shown in Figure 1.

### 3.4. Effect of Wait-Time on Cancer Stage

There was no effect of time to endoscopy on the presence of lymph node positivity or distant metastatic disease at the time of diagnosis (see Table 3 for analysis).

## 4. Discussion

### 4.1. Wait-Times for GI Office Assessment

The mean wait-time from receipt of referral to first endoscopic procedure for subjects in this study was 94 days; 59% of subjects waited longer than 60 days for their first colonoscopy. Patients presenting with alarm signs and symptoms waited 86 days. As per the Canadian Association of Gastroenterology Wait Time Consensus Group, patients who present with iron-deficiency anemia, a change in bowel habits, bright red blood per rectum (BRBPR), positive FOBT or FIT should have an endoscopic procedure within 2 months [3]. In the majority of the time, therefore, patients in this study were not assessed in accordance with recommended guidelines.

There was a significant difference in wait-times between those patients who had either signs or symptoms at the time of referral to gastroenterology, compared to those who were referred for other reasons and were otherwise asymptomatic. This suggests that the gastroenterologists at SPH were appropriately giving priority to those who had alarm symptoms at the time of referral.

Given that the population of interest in this study was diagnostically undifferentiated patients seen nonurgently in the outpatient setting, we attempted to exclude those patients who were seen urgently. However, of the included subjects, 16 (7%) had endoscopy within 14 days, which suggests they were likely expedited, although this was not documented in the chart. Wait-lists for endoscopy at the involved GI clinic are likely even longer than the current results would suggest.

The BC Colorectal screening program commenced at the end of 2013 and offers screening for all asymptomatic adults between the ages 50 and 74. This will likely impact the wait-times for endoscopy in BC given that those patients who have a positive FIT will require endoscopy. Presently, even the Colon Cancer Screening Program has long waiting lists for endoscopic assessment. The majority of patients who underwent colonoscopy in this study had symptoms; only 9% of subjects were asymptomatic and referred for routine screening. Most of our subjects, therefore, were average-risk patients with alarm signs and symptoms of possible CRC; they are a different patient population from those patients who are asymptomatic and referred for endoscopy based on a positive FIT screen. We can expect, therefore, that the number of patients requiring endoscopy to rule out CRC will increase significantly with the addition of referrals from the BC Colorectal screening program. It is reasonable to infer that wait-times for endoscopy are likely to increase in the near future.

### 4.2. Surgery Wait-Times

At present, there are no national benchmarks for cancer surgery wait-times. A number of different published papers have suggested maximum wait-times from primary care referral to surgery; recommended maximum wait-times range from 2 months [6] to 3 months [7]. In the present study, patients waited an average of 147 days or almost 5 months from initial referral to surgery; the majority of the delay occurred in the wait from referral to endoscopy. In a recent Canadian study of over 10,000 patients, a benchmark of 28 days from consultation with surgeon to surgery was chosen; median wait-times were found to be 31 days [8]. In our study, patients waited an average of 29 days.

### 4.3. Wait-Time and Cancer Stage

There was no significant effect of wait-time on the stage of cancer at the time of diagnosis in this study. However, these results should be interpreted with caution given the small number of observations. A larger sample size may be required to assess this more accurately.

Other studies have found similar results. Arbman et al. found that there was no effect of delay in diagnosis with stage of colon cancer, although there was a significantly larger proportion of Dukes' A tumours in patients with rectal cancer with a delay of less than a month between start of symptoms and diagnosis [9]. In another study, delay of treatment was strongly associated with advanced stage of rectal cancer, but not of colon cancer [10]. In an interesting meta-analysis of 5402 patients with CRC, there was no association between delay to diagnosis and stage of cancer [11]. However, when cancers of the colon and rectum were analyzed separately, there was a nonsignificant trend that suggested opposing associations: more delay to diagnosis was associated with an earlier stage of colon cancer, and more delay was associated with a later stage of rectal cancer [11].

Stage of CRC at the time of diagnosis is one of the main determinants of prognosis [12]. Several studies have found no effect of delay in diagnosis on outcome. In a multivariate analysis, Roncoroni et al. found that there was no independent effect of delay in diagnosis in 5-year survival and disease-free survival in a prospective study of 100 patients affected by CRC [12].

This may be due to the fact that the initial symptoms of colon cancer are often vague [13]. In a recent survey, many patients with CRC delayed seeing a physician for more than a month because they thought their symptoms were not serious. In the current study, there was no relationship between type of symptom and stage of cancer; this has been noted in other studies [14]. Delays in diagnosis are therefore likely due in part to the nonspecific nature of symptoms with CRC and the variability of presentation between patients [14]. All patients with symptoms of any kind should therefore be seen for endoscopy in a timely manner.

## 5. Future Research

With the onset of the BC Colon Cancer Screening Program, there will likely be an increase in the number of referrals for endoscopy for rule-out of colorectal neoplasia, and we anticipate that this will impact the number of referrals for endoscopy. It would therefore be of value to repeat the current study to evaluate the impact of the new screening program on wait-times to endoscopy.

## 6. Limitations

As this is a retrospective chart review, the main limitations of this study related to the quality and availability of the health records information. Barriers to data extraction included incomplete documentation, missing charts, information that was unrecoverable or unrecorded, and difficulty in interpreting information found in the documents.

The current study evaluated the wait-times for endoscopy at the PGA office only and, as such, is not generalizable to other specialists providing endoscopy procedures within the Medical Services Plan of BC.

## 7. Conclusion

In keeping with other recent Canadian wait-time studies, the current study finds that average-risk patients referred for rule-out of CRC continue to wait too long for endoscopy. At a local office level, there may be strategies that could be implemented to expedite patients, such as booking direct to endoscopy in those patients referred with alarm symptoms.

## Figures and Tables

**Table 1:** Summary of patient and cancer characteristics for included subjects. The majority of patients were male. The majority of patients had alarm symptoms at the time of referral to the SPH gastroenterologists. The location of cancer was distal in 60% of patients, with either CRC in the rectum or sigmoid colon on surgical pathology. Most patients had no evidence of local nodal invasion (as determined by pathology) and no evidence of distant metastatic disease (as determined by imaging) at the time of diagnosis.

Characteristic	*N* (percent)
Sex	
Male	101 (59)
Female	145 (41)
Location of cancer	
Rectum	73 (30)
Sigmoid	74 (30)
Descending	18 (7)
Transverse	21 (9)
Ascending	27 (11)
Cecum	28 (11)
Two primaries	5 (2)
Symptom on presentation	
Asymptomatic	21 (8)
BRBPR	73 (30)
Change in bowel habits	39 (16)
Anemia	27 (11)
Positive FIT/FOBT	86 (35)
Node positivity	
Positive	94 (38)
Negative	139 (57)
Could not be determined	13 (5)
Distant metastatic disease	
Present	20 (8)
Absent	213 (87)
Could not be determined	13 (5)

BRBPR: bright red blood per rectum.

FIT: fecal immunochemical test.

FOBT: fecal occult blood test.

**Table 2:** Calculated wait-times (mean, median, and range) for office visits and procedures for included subjects for several time periods from initial referral to the PGA office through to surgery by a colorectal surgeon. Subjects were excluded from the calculations if they were not referred to a surgeon or if they declined surgery. Subjects were also excluded from the time to surgery calculations if they had preoperative radiation therapy or chemotherapy. Subjects waited a mean of 63 days to see a GI and 94 days for their first endoscopy procedure. The subset of subjects who had alarm signs or symptoms (BRBPR, anemia, positive FIT/FOBT, or change in bowel habits) at the time of referral waited a mean of 86 days for endoscopy. Total wait-time from referral through to surgery was a mean of 146 days.

Time period	*N*	Wait-time in days		
		Mean	Median	(Range)
Referral to GI office visit	246	63	49	(0–422)
Referral to first endoscopy	246	94	76	(1–428)
Referral to first endoscopy in subjects with symptoms	225	86	70	(1–428)
Endoscopy to office visit with surgeon	221	19	13	(0–221)
Office visit with surgeon to surgery	179	29	24	(0–187)
Total wait-time: referral to surgery	191	146	123	(8–500)

GI: gastroenterologist.

**Table 3:** Effect of endoscopy wait-time on cancer stage. The results of an analysis of the effect of wait-time from receipt of referral to endoscopy on the presence of node positivity and distant metastatic disease are shown in the table. All included subjects were divided into two groups based on whether the time from referral to endoscopy was within guidelines (less than 60 days) or outside of guidelines (greater than 60 days). *p* values were calculated via chi-squared analysis. Of the total 246 included subjects, 94 subjects had cancer in resected lymph nodes; 21 subjects had evidence of distant metastases on staging CT scan. There was no significant effect of longer wait-times on presence of diseased lymph nodes or distant metastases.

Time to endoscopy	*N*	Node positivity (%)	*p* value	Distant mets (%)	*p* value
Within guidelines (<60 days)	102	42 (41)	0.42	12 (12)	0.13
Outside guidelines (>60 days)	144	52 (36)		9 (6)	

mets: metastases.
